# Supplementary material for: CRMS/CFSPID Subjects Carrying D1152H CFTR Variant: Can the Second Variant Be a Predictor of Disease Development?
Source: Diagnostics (Basel). 2020 Dec 12;10(12):1080. doi: 10.3390/diagnostics10121080 (PMC7764752; doi:10.3390/diagnostics10121080)
Supplement: Supplementary file 1 [file diagnostics-10-01080-s001.pdf]

Table S1. CFTR genotype, IRT, first and last sweat chloride in the 43 CRMS/CFSPID subjects carrying D1152H variant.

CFTR Genotype		IRT	First SC	Last SC	Final Diagnosis	Group
**First Variant**	**Second Variant**	**ng/mL**	**mmol/L**	**mmol/L**		
D1152H	F508del	61	39	32		A
D1152H	R553X	65	39	45		A
D1152H	G542X	67	34	71	CF	A
D1152H	F508del	68.39	46	46		A
D1152H	W1282X	70.3	39.3	39.3		A
D1152H	F508del	73	14	26		A
D1152H	2789+5G>A	74.2	49	49		A
D1152H	R1006H	122.4	27.9	27.9		A
D1152H	R1006H	163.8	33.1	33.1		A
D1152H	R1006H	70.3	26.8	26.8		A
D1152H	R1006H	96.5	27.9	27.9		A
D1152H	F508del	75	28.6	28.6		A
D1152H	F508del	76	25	11		A
D1152H	T338I	76.9	39.4	39.4		A
D1152H	3849+10KbC>T	81.4	18.15	34		A
D1152H	F508del	91.3	25	25		A
D1152H	F508del	93	23	29		A
D1152H	F508del	99	15	15		A
D1152H	F508del	100.4	29	48.9		A
D1152H	R1158X	101	20	20	CFTR-RD	A
D1152H	Y849X	103	14	14		A
D1152H	L732X	108	21	21	CFTR-RD	A
D1152H	2789+5G>A	110.1	32.5	32.5		A
D1152H	F508del	116	12	24	CFTR-RD	A
D1152H	R1158X	132	22	35		A
D1152H	F508del	143	36	36		A
D1152H	F508del	147	36	21		A
D1152H	R347H	53	14	15		A
D1152H	S1426F	64	10	12		B
D1152H	5T12TG	66	11	11		B
D1152H	M952T	66.9	27.02	24		B
D1152H	D1152H	68	33	33		B
D1152H	5T-TG11	70	22	52		B
D1152H	L977F	70	10	10		B
D1152H	R1070Q	76	17	16		B
D1152H	L977F	73.9	33.56	41		B
D1152H	D1152H	79.3	30.1	30.1		B
D1152H	L749L	79.9	59	15.5		B
D1152H	[p.C832G,5T-12TG]	81	27	17		B
D1152H	5T12TG	82	7	8		B
D1152H	D1152H	95	15	8		B
D1152H	L977F	101	29.5	29		B
D1152H	5T11TG	104	32	13		B

Abbreviations: CFTR: Cystic fibrosis transmembrane conductance regulator; CF: Cystic Fibrosis; CFTR-RD: CFTR Related Disorder; IRT: immunoreactive trypsinogen; SC: sweat chloride.

Table S2. List of *CFTR* variants *in trans* with the D1152H variant.

CF Causing §			
**Legacy name**	**HGVS Nucleotidic Notation**	**HGVS Aminoacidic Notation**	**Frequency N (%)**
F508del	c.1521_1523delCTT	p.Phe508del	12 (27.9%)
R1066H	c.3197G>A	p.Arg1066His	4 (9.3%)
2789+5G>A	c.2657+5G>A	-	2 (4.6%)
R1158X	c.3472C>T	p.Arg1158*	2 (4.6%)
G542X	c.1624G>T	p.Gly542*	1 (2.3%)
R553X	c.1657C>T	p.Arg553*	1 (2.3%)
T338I	c.1013C>T	p.Thr338Ile	1 (2.3%)
L732X	c.2195T>G	p.Leu732*	1 (2.3%)
R347H	c.1040G>A	p.Arg347His	1 (2.3%)
W1282X	c.3846G>A	p.Trp1282*	1 (2.3%)
3849+10kbC>T	c.3717+12191C>T	-	1 (2.3%)
Y849X	c.2547C>A	p.Tyr849*	1 (2.3%)

Non CF-Causing § (Varying Clinical Consequences; Unknown Significance)			
**Legacy Name**	**HGVS Nucleotidic Notation**	**HGVS Aminoacidic Notation**	**Frequency N (%)**
L997F [a]	c.2991G>C	p.Leu997Phe	4 (9.3%)
(TG)12T5 [b]	c.[1210-34TG [12];1210-12T[5]]	-	3 (6.9%)
D1152H [b]	c.3454G>C	p.Asp1152His	2 (4.6%)
(TG)11T5 [b]	c.[1210-34TG[11];1210-12T[5]]	-	2 (4.6%)
L749L [d]	c.2245C>T	p.Leu749Leu	1 (2.3%)
M952T [c]	c.2855T>C	p.Met952Thr	1 (2.3%)
R1070Q [b]	c.3209G>A	p.Arg1070Gln	1 (2.3%)
S1426F [d]	c.4277C>T	p.Ser1426Phe	1 (2.3%)

§ variants are classified according to CFTR2 mutations database; [a] variant non CF-causing (that however can cause CFTR-RD [35–37]); [b] variant with varying clinical consequences; [c] variant with unknown significance; [d] variant not present in CFTR2 mutations database.
